# Chronic lifestyle diseases display seasonal sensitive comorbid trend in human population evidence from Google Trends

**DOI:** 10.1371/journal.pone.0207359

**Published:** 2018-12-12

**Authors:** Jai Chand Patel, Pankaj Khurana, Yogendra Kumar Sharma, Bhuvnesh Kumar, Sugadev Ragumani

**Affiliations:** Defence Institute of Physiology and Allied Sciences, Defence Research and Development Organization, Timarpur, Delhi, India; Kyushu Institute of Technology, JAPAN

## Abstract

Seasonal and human physiological changes are important factors in the development of many diseases. But, the study of genuine seasonal impact on these diseases is difficult to measure due to many other environment and lifestyle factors which directly affect these diseases. However, several clinical studies have been conducted in different parts of the world, and it has clearly indicated that certain groups of population are highly subjected to seasonal changes, and their maladaptation can possibly lead to several disorders/diseases. Thus, it is crucial to study the significant seasonal sensitive diseases spread across the human population. To narrow down these disorders/diseases, the study hypothesized that high altitude (HA) associated diseases and disorders are of the strong variants of seasonal physiologic changes. It is because, HA is the only geographical condition for which humans can develop very efficient physiological adaptation mechanism called acclimatization. To study this hypothesis, PubMed was used to collect the HA associated symptoms and disorders. Disease Ontology based semantic similarity network (DSN) and disease-drug networks were constructed to narrow down the benchmark diseases and disorders of HA. The DSN which was further subjected to different community structure analysis uncovered the highly associated or possible comorbid diseases of HA. The predicted 12 lifestyle diseases were assumed to be “seasonal (sensitive) comorbid lifestyle diseases (SCLD)”. A time series analyses on Google Search data of the world from 2004–2016 was conducted to investigate whether the 12 lifestyle diseases have seasonal patterns. Because, the trends were sensitive to the term used as benchmark; the temporal relationships among the 12 disease search volumes and their temporal sequences similarity by dynamic time warping analyses was used to predict the comorbid diseases. Among the 12 lifestyle diseases, the study provides an indirect evidence in the existence of severe seasonal comorbidity among hypertension, obesity, asthma and fibrosis diseases, which is widespread in the world population. Thus, the present study has successfully addressed this issue by predicting the SCLD, and indirectly verified them among the world population using Google Search Trend. Furthermore, based on the SCLD seasonal trend, the study also classified them as severe, moderate, and mild. Interestingly, seasonal trends of the severe seasonal comorbid diseases displayed an inverse pattern between USA (Northern hemisphere) and New Zealand (Southern hemisphere). Further, knowledge in the so called “seasonal sensitive populations” physiological response to seasonal triggers such as winter, summer, spring, and autumn become crucial to modulate disease incidence, disease course, or clinical prevention.

## Introduction

Seasonal changes in the environment have huge impact in all species and their adaptation is critical for their survival [1,2]. Indeed, several clinical studies have been conducted in different parts of the world, clearly indicating that certain groups of population are highly subjected to seasonal triggering, and their maladaptation can possibly lead them to several seasonal sensitive disorders/diseases [3–7]. Even though seasonal variations affect the entire human physiological systems, the cardio-vascular systems, cardio-respiratory systems, and circadian rhythms are most sensitive to these seasonal changes. In recent days, there is an impressive pattern of seasonal rhythm in hospitalizations of patients with cardio-vascular and cardio-respiratory diseases or disorders, with a notable increase in winter [8–11].

However, the study of genuine seasonal impact on these diseases is highly complex to decipher due to two main reasons: (i) apart from seasonal changes, a number of other environment and lifestyle factors which directly affect these diseases onset and severity needs to be controlled, such as air pollution, geographic location, ethnicity, physical exercise, social interactions and so on [7,12–19]. For example, the seasonal pattern in the incidence of asthma attacks onset and severity was highly influenced by several environment and lifestyle factors [20–23]; (ii) this severity was further enhanced and was proportional to the number of co-occurrence of diseases (comorbid diseases). For example, asthma is highly comorbid with many other diseases such as cardio cerebrovascular diseases, obesity, hypertension, diabetes, psychiatric conditions, neurological disorders, gut and urinary disorders, cancer, respiratory problems, and so on [24]. Among these, identifying the most significant seasonally comorbid diseases of asthma is crucial. Lack of reports on these seasonal linkages restricts the implementation of human seasonal adaptation in clinical environment.

High altitude (HA) elevations are ranging from 3,000 to 5,000 meters and the partial oxygen pressure is only about 70% of the value at sea-level [25]. Firstly, in HA, humans undergo significant physiological changes, mainly in cardio respiratory and cerebral functions, leading to the temporary physiological human adaptation called acclimatization [26]. HA disorders encompass the pulmonary and cerebral syndromes that occur in non-acclimatized individuals shortly after rapid ascent to HA [27]. The maladapted human population develop several HA disorders like acute mountain sickness (AMS), high altitude pulmonary edema (HAPE), and high altitude cerebral edema (HACE). Many seasonal symptoms and disorders act as the predisposing factors of HA disorders. For example, patients with asthma, obesity, and hypertension were more prone to develop AMS [28–30]. The present study tried to address both the limiting factors using the high altitude linked diseases and disorders.

The internet is now an important source of health information for millions of people worldwide, which makes google search queries a valuable source of information for the collection health trends [31]. In recent years, “Google Trends” (GT) is gaining momentum to assess seasonal changes in diseases because it offers search information about a disease for long period of Jan 2004 to the present week. For example, the seasonality in Cold Flu, Influenza, Urinary Tract Infection, Ankle swelling, and Vitamin D were accurately estimated using Google Trends [32–35]. Furthermore, GT provides the total volumes for requested diseases terms, normalized in a way that countries of different size can be compared [31]. Importantly, GT was used to study the precise interaction of search terms effect using combined diseases terms (https://support.google.com/trends/answer/4359550?hl=en-GB&ref_topic=4365530). The present study utilized GT to estimate the seasonality in the diseases as well as to measure the comorbid associations among them.

The current study had the following research objectives: (1) to decipher the genuine seasonal diseases using the HA related diseases and disorders from the literature mining; (ii) to conduct disease ontology based semantic similarity network and community detection approach to find the most likely seasonal comorbid diseases; (iii) to evaluate seasonal comorbid diseases, the GT search terms volume of the respective diseases were subjected to rigorous time series analysis. The main outcome of the analysis includes: (a) reveals that a considerable number of human populations is severely subjected to annual seasonal variations; (b) highlights of observed high seasonal rhythm among hypertension, obesity, asthma, and fibrosis diseases are widely spread among the human population. To our knowledge, this is the first study of its kind to examine the possibility of existence of seasonal association in comorbid life style diseases in human population.

## Materials and methods

### Data mining

**Fig 1:**
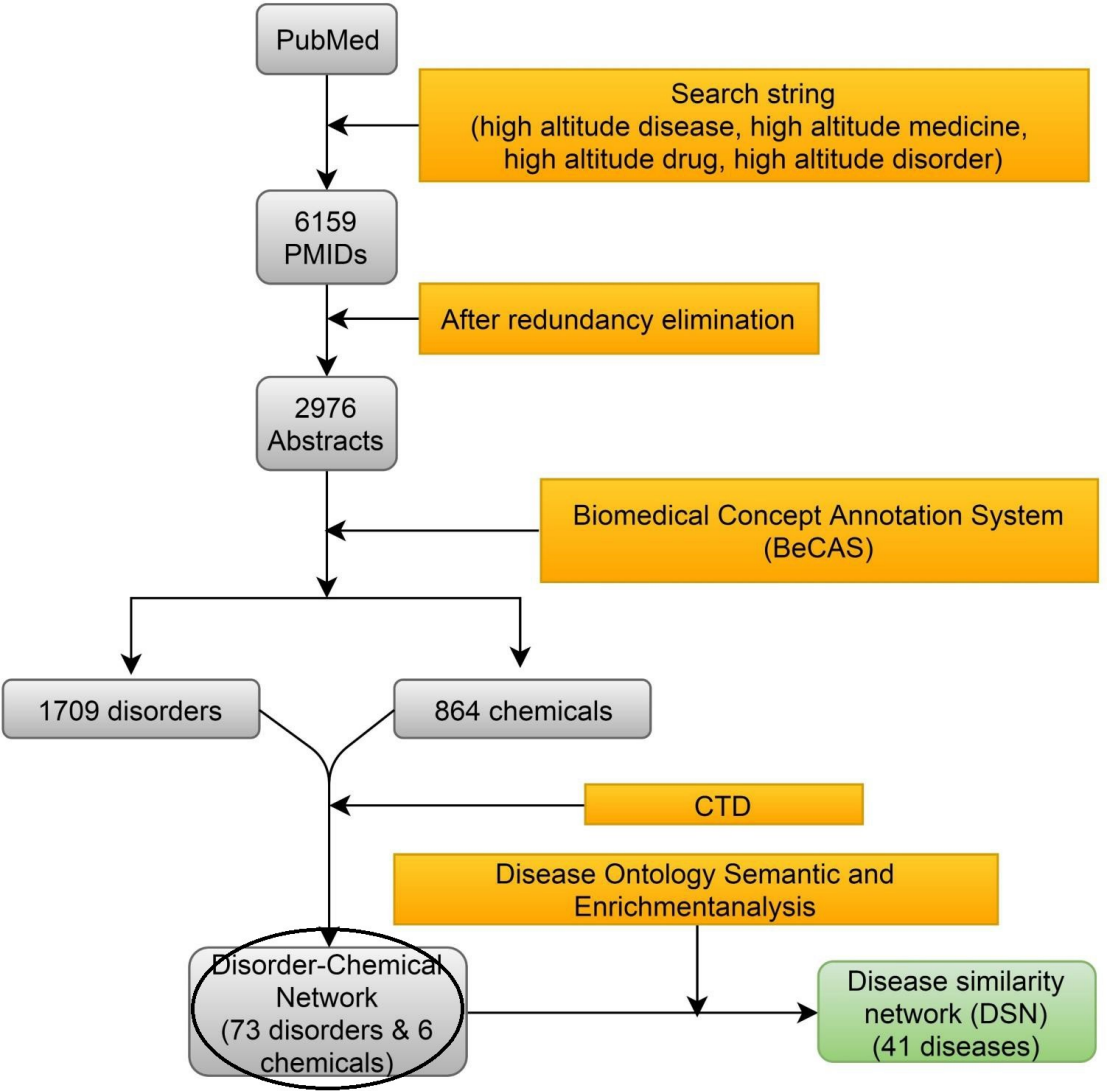
The workflow pipeline followed to build the disease similarity network (DSN) of high altitude related diseases. **Step 1:** PubMed Central was queried for high altitude related abstracts. **Step 2:** All high altitude abstracts were collected and redundancies were removed. **Step 3:** The Biomedical concept annotation system (BeCAS) was used to extract the high altitude related diseases/disorders and chemicals from the collected abstracts. **Step 4:** The relations among diseases and chemicals from Comparative Toxicogenomics Database (CTD) were used to construct the Disorder-Chemical Network. **Step 5:** Disease ontology based semantic similarity-based score was used to construct the Disease similarity network (DSN).

The aim of this study is to contribute a novel association generation among high altitude related diseases/disorders, symptoms, drugs, and medicines. Fig 1 illustrates various steps involved in the data collection and analysis. The first step in the study was to retrieve all relevant papers related to the topic of interest, i.e., high altitude. Abstracts that contain the most important and concise diseases/disorders and symptoms linked to high altitude were then chosen to be examined. Although the full-text analysis is more informative, the abstract based disease terms analysis has added advantages. Mainly, the analysis is more informative and concise. Also, it is faster to compute with reduced noise level. Keeping in view, a PubMed medical subject headings (MeSH) terms query was used to collect all possible high-altitude related abstracts. PubMed was queried for four types of key terms related to high altitude including: “High altitude disease”, “High altitude disorder”, “High altitude medicine” and “High altitude drug”. PubMed abstracts related to human studies were extracted by using the search filter option “Human”. The total number of Pubmed Identification numbers (PMIDs) retrieved from the four key terms was 6159 as on Jan 2017. The redundant PMIDs were reduced resulting in 3513 unique PMIDs. The abstracts of these unique PMIDs were fetched using “PubMedWordcloud” of R console [36]. Overall, 2976 abstracts were ready for knowledge discovery.

### Entity selection and extraction

The next step is to extract the entities such as diseases/disorders, symptoms, drugs and medicines associated with high altitude. The association of the entities with high altitude means any loose relation that covers biological, biomedical or health related interest. To study the association, an online application programming interface (API) called BioMedical Concept Annotation System (BeCAS) was adopted [37]. This API is widely used to identify biomedical concepts in text [38–40]. BeCAS employs a standard pipeline that consists of sentence boundary detection, tokenization, lemmatization, part of speech tagging, and chunking and abbreviation disambiguation. 2976 abstracts were supplied as input to BeCAS. Among the 11 default entities of BeCAS, the term fetching for diseases and disorders entities was carried out. GDep, a dependency parser with the option of domain adaptation using unlabeled data of the target domain, was used to achieve most of the pre-procession steps in BeCAS. For entity recognition, BeCAS uses UMLS extended with a series of more specialized dictionaries such as the Joint chemical dictionary (Jochem).

### Benchmark high altitude diseases/disorders

First, the high altitude related chemicals and their target diseases/disorders were used to generate a bipartite network. The known associations between chemicals (or equivalently, drugs) and disorders or its descendants were collected from Comparative Toxicogenomics Database (CTD) in Feb 2017 [41]. In this study, the researchers mainly extracted curated associations of chemical-diseases from CTD to ensure the strong association between chemicals (or equivalently, drugs) and disorders or its descendants. The size of the nodes is based on the frequency of the diseases that were occurring in the resulting dataset composed of the 2976 abstracts. The Gephi open source graph visualization software tool is used to develop a graphic representation of the extracted interaction network [42]. Analysis of the generated network was carried out by Cytoscape network analyser [43]. Diseases that missed their links with drugs/chemicals were left out from their disease module. Drug-disease network property and node size (frequency in literature) were used to narrow down the significant high altitude diseases/disorders and chemicals/drugs.

### Clustering disease-disease networks

The disease-disease association among the benchmark high altitude diseases/disorders was depicted in the form of network. The association was established using the DOSE package for R based on computing semantic similarities among Disease Ontology (DO) terms associated with every disease pair [44]. The DO score in correlation matrix ranged between 0–1. Various types of cut-off techniques such as clustering based, score based, percentage based, etc., were employed. In the execution, the threshold value as topmost three edge score (even after transposition of matrix) arising from a single node was used. The clustered disease modules were constructed using fast greedy, edge betweenness, spin glass and walk trap based clustering methods [45–48]. These different disease network modules were superimposed with each other to identify the overlapped cluster disease modules. The DO score-based disease mapping was carried out to detect disease outliers.

### Google trend data collection

**Fig 2:**
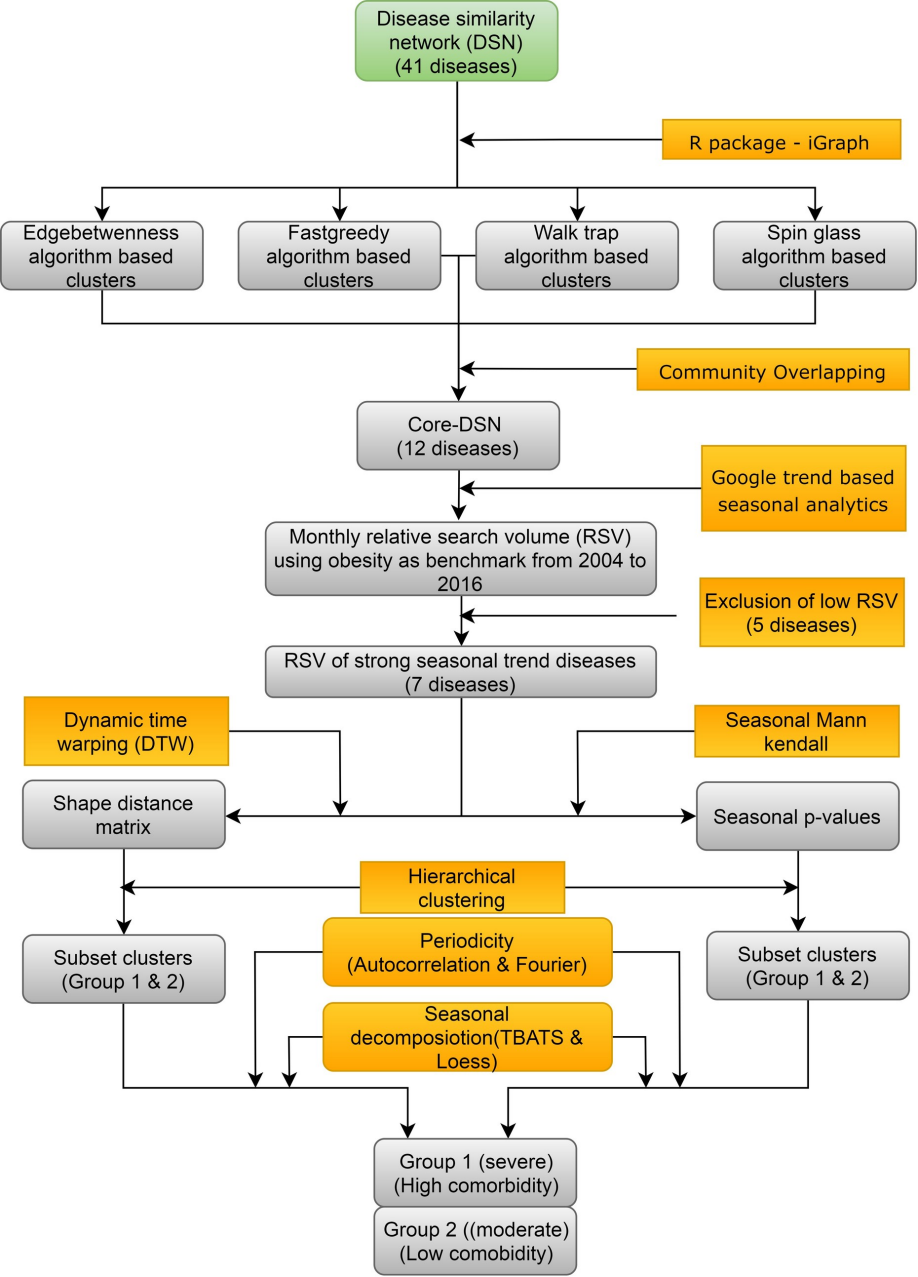
The pipeline for prediction of comorbid seasonal diseases from DSN. The pipeline starts from the DSN corresponds to step 5 in Fig 1. **Step1:** the DSN was clustered using four community clustering algorithms of igraph program. **Step 2:** These community clusters were overlapped to predict the 12 diseases (core-DSN). **Step 3:** The monthly Google RSV were collected for the 12 diseases with obesity as the benchmark disease. **Step 4:** The low RSV diseases were removed, and the remaining diseases were subjected to seasonal Mann-Kendall and Dynamic time wrapping methods. **Step 5:** Based on their seasonal trend p-values and shape distance matrix clustering the diseases were divided into two groups severe (high) and moderate (low). **Step 6:** Their seasonal trends were further verified using seasonal decomposition analysis by TABTS and LOESS. The periodicity was analyzed using Autocorrelation and Fourier series analysis.

Fig 2 shows the various steps in the analysis of GT. GT is used to study the temporal trends in web search using monthly and weekly Relative Search Volume (RSV). In the query, “all categories” and “all types of web search” were used, which are the default setting of GT. We searched “worldwide”, using the default settings, for the time period of Jan 2004 to Dec 2016. The rationale behind the selection of default option was to include wide variety of web resources such as image, you tube, news and Google shopping in the GT web search. The “worldwide” search option allowed us to collect RSV from almost 250 political regions, including sovereign as well as dominion states with most of them (162 countries) in the northern hemisphere (NH), covering almost 90% of our human population. RSV is calculated based on how often search terms entered in Google relative to the total search volume in a specific region. These RSV were also collected with and without a reference disease such as obesity in our case. The rationale behind the selection of obesity as the reference disease will be explained in the discussion section under the sub heading “Widespread seasonal comorbid rhythm in the severe SCLD”. The search volume of the term was normalized by dividing an unrelated common web search query. This normalization process of GT was compensated for population sizes, increased sensitivity in detection, and allowed the direct comparison of the search volume from countries/cities and different diseases. The RSV scaled between 0 and 100, with 100 being the highest search proportion per week. GT uses internet protocol (IP) addresses from server logs to assign the origin of web search queries. The monthly and weekly RSV for 12 diseases was downloaded in *.csv format with and without reference diseases for the period of 2004 to 2016 across the world. It should be noted that the relative medical terminologies, similar medical conditions and related search strings excluded in the GT search. In our opinion, such a comprehensive analysis is beyond the scope of the current study. Our GT search terms were limited to English speaking population, because this population is widely spread in world. Furthermore, two English speaking countries, United States of America (USA) and New Zealand (NZL) were geographically selected above 23.5°N and below 23.5°S to represent Northern and Southern hemispheres (SH) seasonal changes respectively. In both the countries, RSV of the four severe seasonal comorbid diseases namely Asthma, Hypertension, Obesity and Fibrosis were collected with obesity as the reference for the period from January 2004 to December 2016.

### Time series analysis

Data processing and statistical analysis were carried out using Trend package of R [49]. Time series related figures were produced using the TSA package [50]. The seasonal data component decomposition of each search term time series was carried out by local regression (LOESS) [51]. The Mann-Kendall and seasonal Mann-Kendall trend tests were used to detect overall trends significantly larger than the variance in the data for each search term (α = 0.05). To determine the significant seasonal components, an exponential smoothing state space model with Box-Cox transformation, trend, and seasonal components (TBATS) were fitted to the data using forecast package [52]. The shape based distance matrix analysis was carried out to check out the distance based on coefficient-normalized cross-correlation which reflects a time series clustering with dynamic time warping optimization [53]. This algorithm first calculates the Z-score to normalize the matrix, and forms a cross-series correlation [54]. After getting final correlation matrix, hierarchical clustering (using pvclust) was used to obtain possible groups [55]. Further, autocorrelation was observed in the disease group showing strong seasonality. Autocorrelation gives an idea about the cyclic pattern present in the data. Fast Fourier Transform (FFT) based periodograms were produced to identify key seasonal cycles in the data from 2004 and 2016 [50]. The breaking down moving average (BMA) with window size of 6 months for the periods October to March and April to September were calculated.

## Results

### High altitude disease-drug network

**Fig 3:**
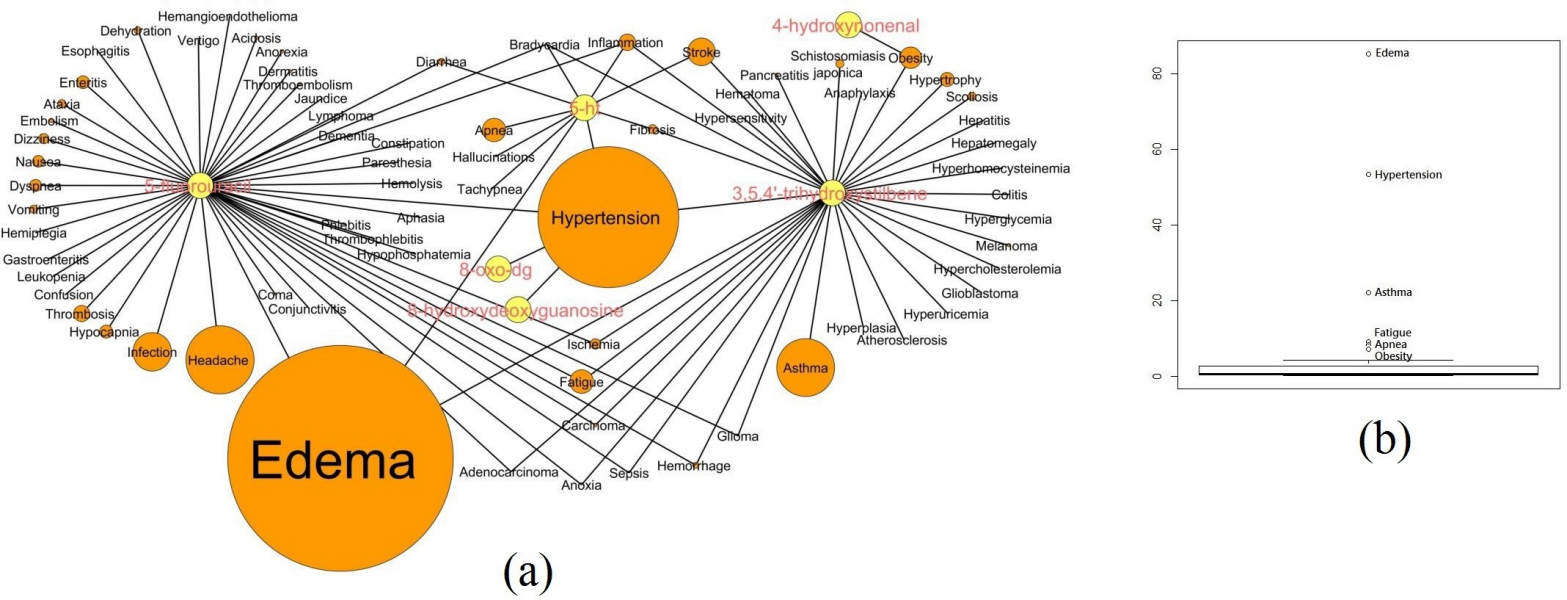
Methods for disease-drug network-based selection for the most relevant high-altitude diseases. **(a)** Link prediction model predict the relationship between high altitude related drugs (yellow) and diseases (orange) based on the clinically reported drug-disease pairs association were obtained from Comparative Toxicogenomics Database (CTD). The node size of each disease term in the network corresponds to their frequency of occurrence in the high altitude related abstracts. **(b)** The outlier plot showed diseases have the high term frequency (n>10) called as bottleneck diseases.

We developed a systems approach to infer benchmark diseases/disorders of high altitude to investigate their seasonality. As mentioned earlier, we have collected comprehensive list of high altitude related disorders/disease and drugs from PubMed. After excluding duplicate medical terms, they were annotated using the most commonly used Medical Subject Headings (MeSH). From 2976 high altitude related abstracts, 1710 diseases and 865 drugs were extracted using the text analytics method BeCAS. In search of closely connected high altitude diseases, the clinically reported drug-disease pairs associations were obtained from CTD [41]. After excluding duplicate drug-disease pairs and unlinked pairs, a high altitude related drug-disease bipartite network with six drugs/chemicals and 73 diseases connected by 95 edges was built (Fig 3A). The node size of each disease term in the network corresponds to its frequency of occurrence in the high altitude related abstracts. The network showed large node size for edema, hypertension, asthma, fatigue, apnea, and obesity diseases/disorders. The outlier plot further showed these six diseases have the high term frequency than the rest of the 67 diseases. Interestingly, edema, hypertension, asthma, fatigue, apnea, and obesity have the high term frequencies (>90 percentile) and node sizes and were termed as key bottleneck diseases of high altitude (Fig 3B).

### High altitude associated diseases community structure analysis

**Fig 4:**
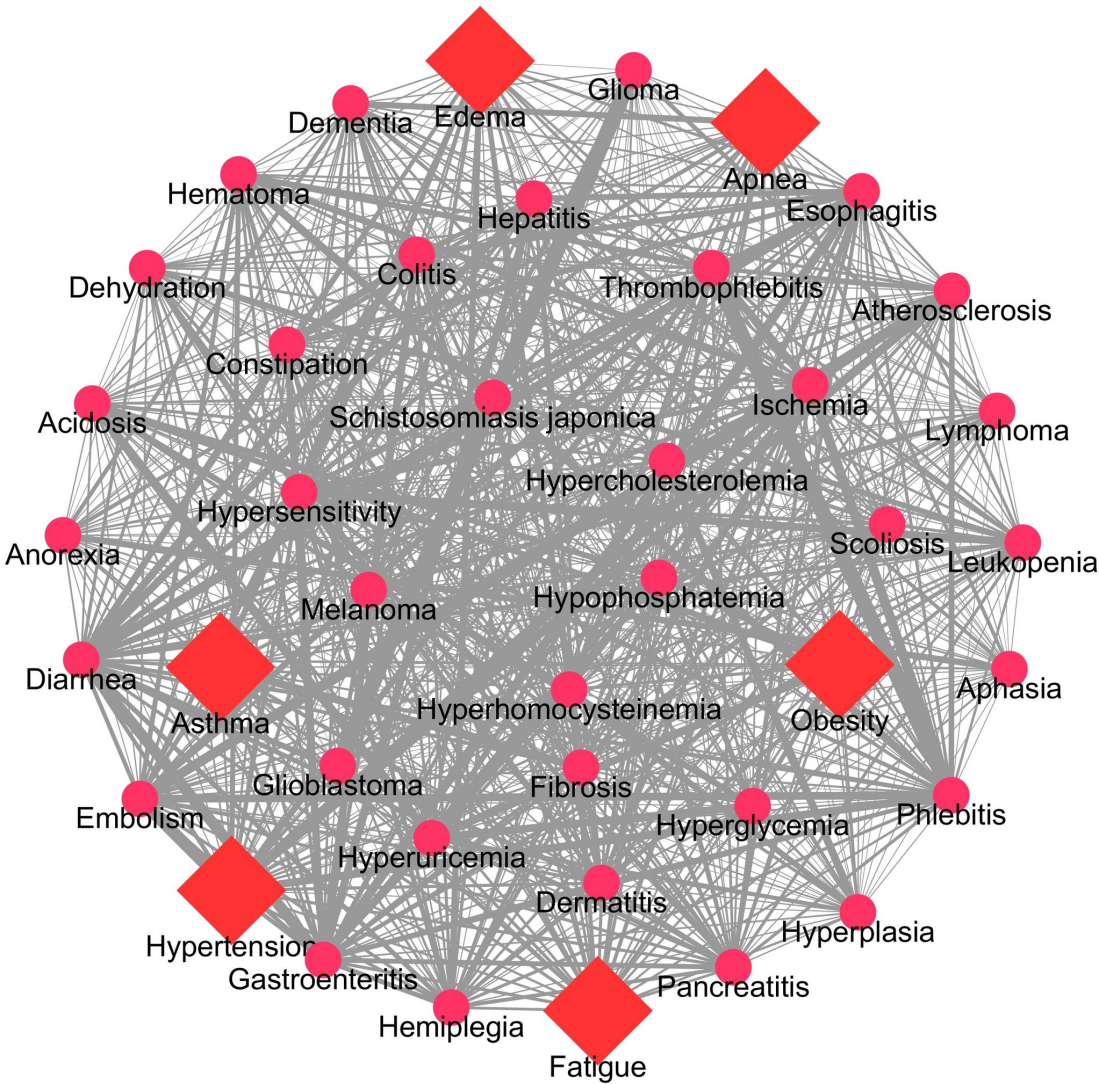
Disease ontology (DO) based semantic similarity disease network (DSN) of high altitude. The disease (red colour square shape) pairs showing the >90 percentile (outliers) literature frequency were used in the construction. The average degree (number of links with other diseases) of all diseases in the disease network is 0.1418 (marked as gray lines). The edge thickness represents the SS score between two diseases. Note that the bottleneck diseases of high altitude in the network are in square shapes rather than circle otherwise.

**Fig 5:**
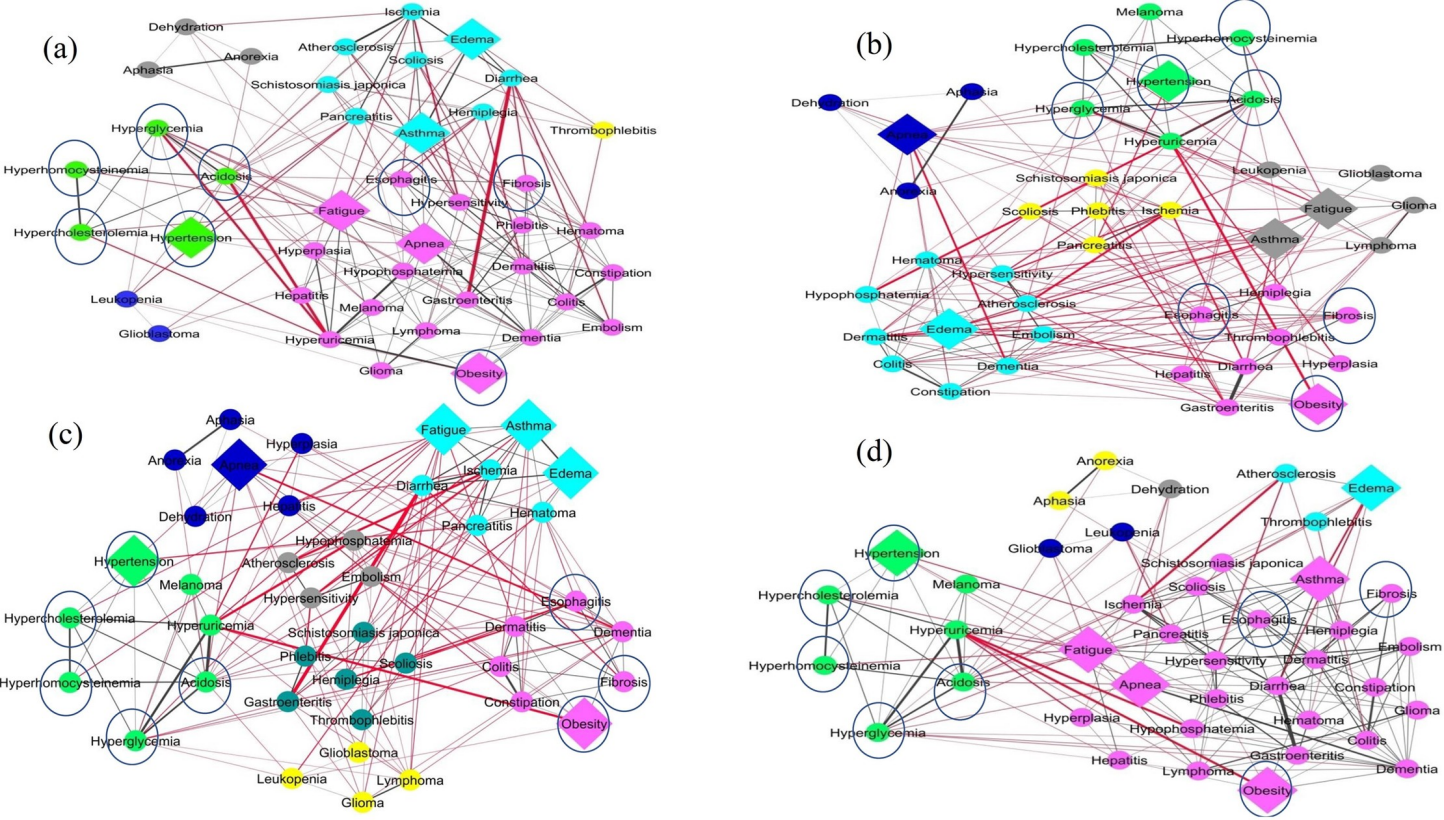
**The DSN subjected to the four community detection algorithms based on the (a) edge betweenness (EB), (b) fast greedy (FG), (c) spin glass (SG) and (d) walk trap (WT) available in the “igraph” package.** Here, we clearly see that among the six bottleneck diseases (square shape edges) only hypertension community (green colour) and obesity community (magenta colour) are tightly maintained by the four community detection algorithms (encircled). Please note that in all the community detection algorithms, the hypertension community associated diseases (acidosis, hypocholesterolemia, hyperhomocysteinemia, and hyperglycemia) formed a major community cluster. Similarly, the obesity community diseases (esophagitis and fibrosis) formed a short community cluster. Whereas, other bottleneck diseases not able to maintain a separate community cluster. Moreover, the overall inter community cluster interactions (red colour edges) are more than intra community cluster interactions (gray colour edges). Visualization of the network was done using cytoscape (Shannon et al., 2003).

**Fig 6:**
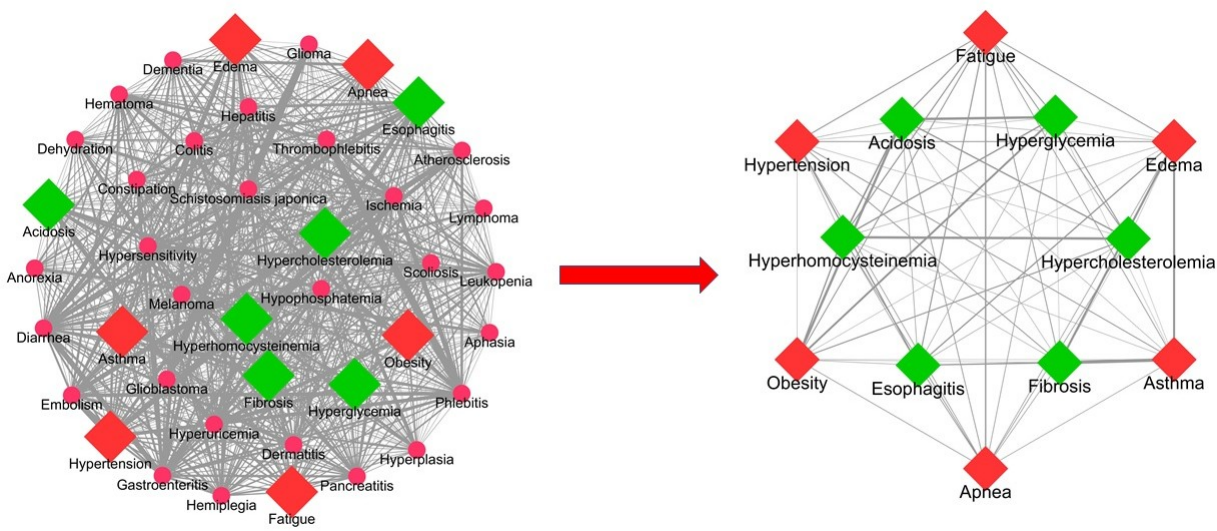
The left-hand side is the DSN of high altitude marked with six bottleneck diseases (square, red colour) and six community diseases (square, green colour). The overall semantic similarity (SS) average score of the DSN is 0.14188. The right-hand side is core DSN network of 12 diseases (six bottleneck and six community diseases). The overall semantic similarity score of the core DSN is 0.19624 and named as “highly comorbid diseases of high altitude”.

To generate a high altitude disease network in which diseases that have similar signs and symptoms were clustered together. Firstly, we generated the disease similarity network (DSN) based on semantic similarities scores among DO terms associated with every disease pair. We computed the pairwise disease-disease semantic similarity matrix from the 73 high altitude related diseases. Among 73 diseases, only 41 diseases (including the six key bottleneck diseases) have disease pair similarity scores with other diseases. The weighted average disease similarity scores were used to generate the DSN. The DSN consisted of 41 nodes and 820 edges (after removing duplicates and self-loop edges) as shown in Fig 4. The DSN network was further subjected to most widely used community detection algorithms, namely- fast greedy (FG), edge betweenness (EB), spin glass (SG) and walk trap (WT) available in the “igraph” package [56]. Each algorithm clustered the DSN into four major disease communities or sub networks (Fig 5). The sub networks identified from the four algorithms were overlapped to uncover the highly associated or possible comorbid diseases. The bottleneck disease associated community clusters were chosen for detailed analysis. Interestingly, in these community clusters, hypertension formed intra-community interactions with hyperglycemia, hyperhomocysteinemia, hypercholesterolemia, and acidosis, whereas obesity formed intra-community interactions with fibrosis and esophagitis. Overall, these six bottleneck diseases and their six community disease pairs formed a core DSN (Fig 6). The core DSN (12 diseases) possessed almost 60% of the similarity score of DSN (41 diseases).

### Monthly google seasonal trend analysis

**Fig 7:**
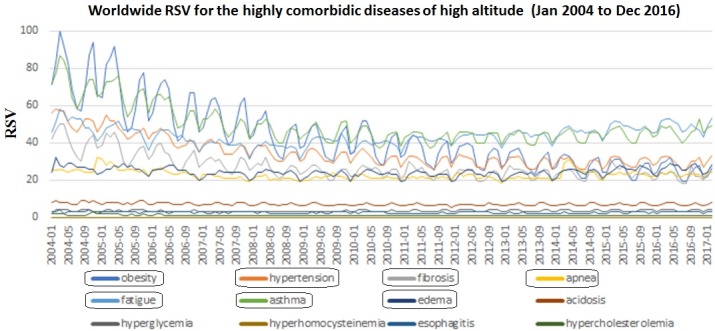
The monthly widespread google trend relative search volume (RSV) collected for the “highly comorbid diseases of high altitude (core DSN)” with obesity (dark blue colour) as the benchmark disease. The average RSV of the core-DSN was diseases more than 20 for the entire period were boxed. These seven diseases were named as “severe seasonal comorbid lifestyle diseases (SCLD)”.

**Fig 8:**
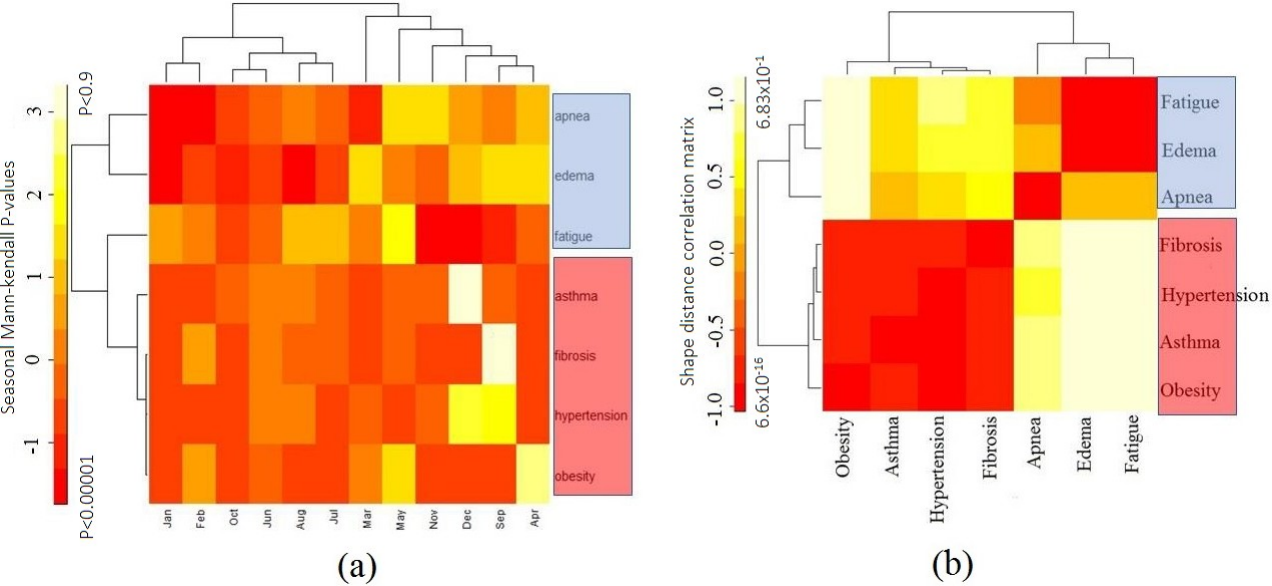
Widespread seasonal sensitive comorbid diseases. **(a)** Seasonal Mann-Kendall p-values of seasonal sensitive life style diseases represented as heat map for the entire period (Jan 2004 to Dec 2016). The hierarchical clustering clearly divided them into diseases with (red box) and without (blue box) season trend. **(b)** Shape based distance matrix scores among the major highly comorbid diseases of high altitude represented as heat map for the entire period (Jan 2004 to Dec 2016). The hierarchical clustering clearly divided them into diseases with (red box) and without (blue box) seasonal pattern matching. Please note that the four diseases (obesity, asthma, hypertension, and fibrosis) in the red boxes have similar seasonal trends and seasonal patterns named as “Group1” diseases (**S1 Table**).

**Fig 9:**
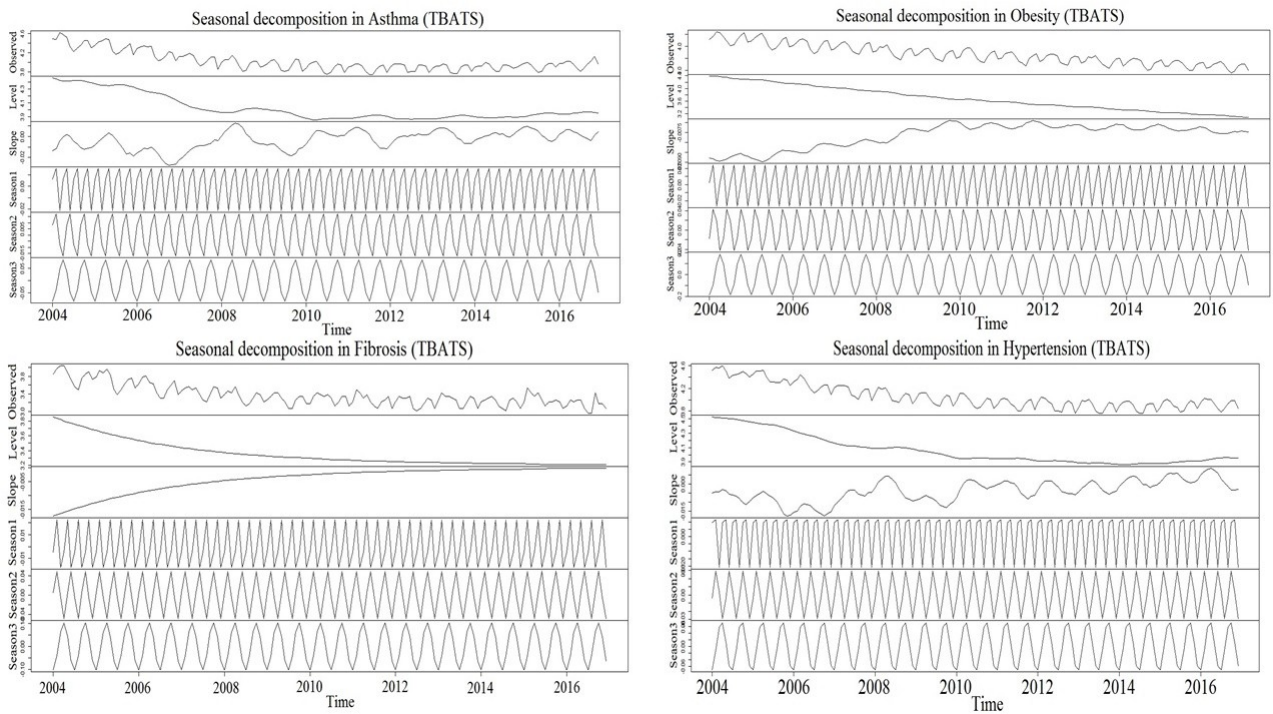
Seasonal and trend decomposition using TBATS for the four group 1 diseases for the monthly collected RSV from Jan 2004–Dec 2016 with obesity as the benchmark disease. Raw observed data were displayed in the top panel as averaged values for all transect points with monthly sampling frequency, followed by level, trend, and seasonal components 1,2, and 3. Scale differs for each of the components, and so relative magnitude was indicated by the gray bars on the left side of the panels.

**Fig 10:**
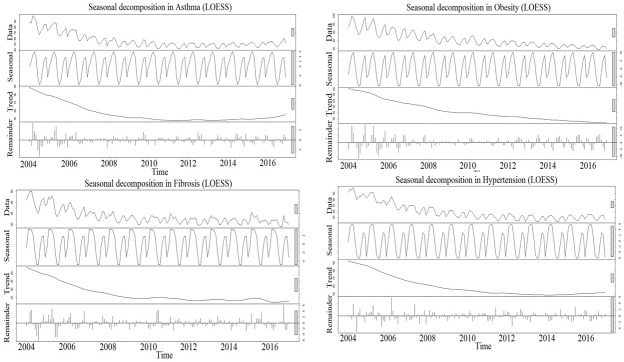
Seasonal and trend decomposition using loess (STL) for the four group 1 diseases for the monthly collected RSV from Jan 2004 –Dec 2016 with obesity as the benchmark disease. Raw data are displayed in the top panel as averaged values for all transect points with monthly sampling frequency, followed by seasonal, trend, and residual components. Scale differs for each of the components, and so relative magnitude was indicated by the gray bars on the right side of the panels.

**Fig 11:**
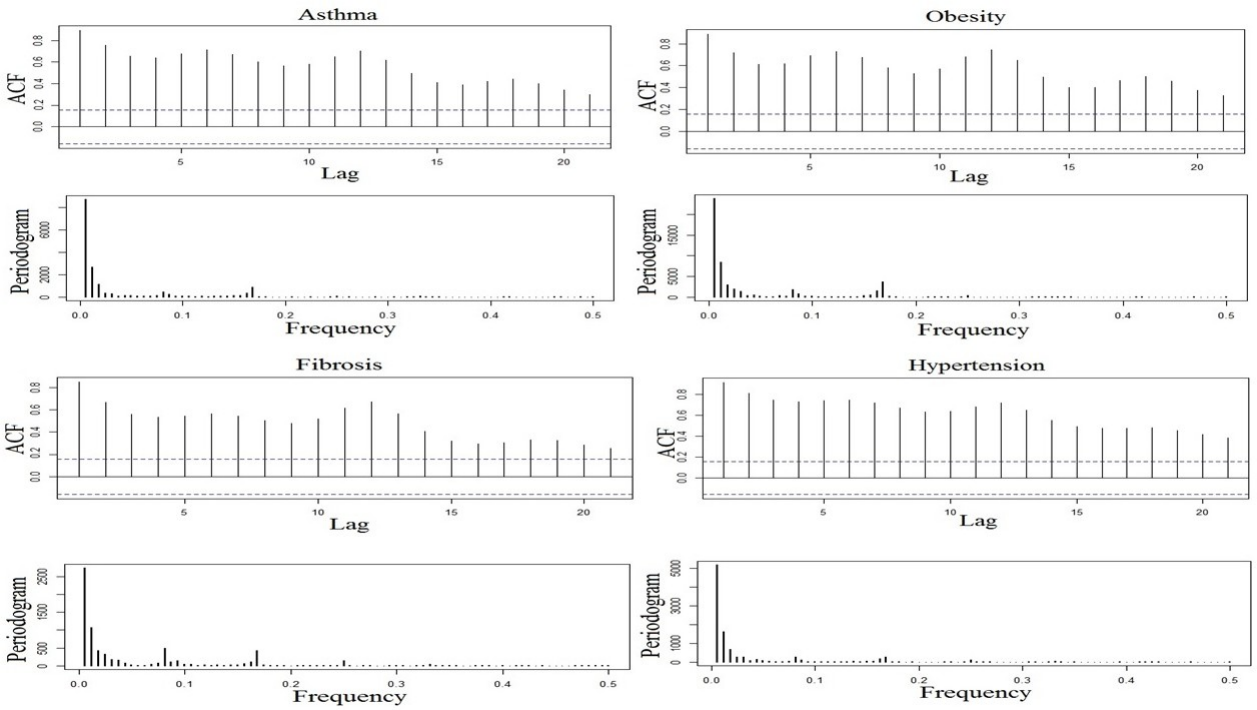
Autocorrelation and periodicity of four group 1 diseases for the monthly collected RSV from Jan 2004–Dec 2016 with obesity as the benchmark disease. Observed data is showing a cyclic pattern in autocorrelation above significant line (dotted) and six months periodicity in all and three months in asthma as well as hypertension (periodogram).

Using obesity as the benchmark keyword, the month wise varying RSV for the 12 diseases (including obesity) were analyzed for seasonal trend in the 2004–2016 period. All the six bottleneck diseases showed high RSV. Except fibrosis, the remaining five community diseases with low RSV were excluded from the trend analysis (Fig 7). The seasonal Mann-Kendall and Mann-Kendall showed positive seasonal trend for asthma, hypertension, obesity, fibrosis, apnea, and fatigue whereas edema showed no seasonal trend (Table 1). The seasonal Mann-Kendall and Mann-Kendall of the month wise RSV of the seven diseases without benchmark keyword also showed similar trends (Table 2). Based on their trend P-values, the seven diseases were clustered into two groups (1 and 2) (Fig 8A, Table 1). The strong seasonal trend in the group 1 diseases was exhibited by TBATS and LOESS analysis (Figs 9 and 10). The auto correlation analysis also revealed the presence of six months periodicity in the group 1 diseases (Fig 11, Table 1). In this direction, we further calculated the optimal shape alignments among the seven diseases RSV using dynamic time warping method. Importantly, the clustering of the shape extraction matrix again divided the seven diseases into two groups (1 and 2) (Fig 8B). All these results strongly suggested that group 1 diseases maintain widely spread seasonal comorbid trend.

**Table 1:** Widespread monthly RSV of GT time series seasonal analysis of the core DSN diseases (Jan 2004 to Dec 2016) with obesity as the reference term. (Complete raw data is given in S1 Table).

Seasonal Comorbid Lifestyle diseases (SCLD) of core DSN	Average RSV	Mann-Kendall Trend Test					Seasonal Mann-Kendall Trend Test				Seasonal Decomposition		Autocorrelation	Periodicity	
		p-value	tau	z	S	varS	p-value	z	S	varS	TBATS	LOESS			
**Asthma**	49.55	<0.0001	-0.44	-8.02	-5274	431995	<0.0001	-8.32	-465	3108	Yes	Yes	Yes	6 months	**Severe comorbid diseases**
**Obesity**	43.01	<0.0001	-0.7	-12.81	-8440	433651	<0.0001	-16.16	-917	3215	Yes	Yes	Yes	6 months	
**Hypertension**	35	<0.0001	-0.63	-11.4	-7500	432005	<0.0001	-13.71	-767	3122	Yes	Yes	Yes	6 months	
**Fibrosis**	27.22	<0.0001	-0.54	-9.71	-6383	431473	<0.0001	-11.98	-669	3108	Yes	Yes	Yes	6 months	
**Fatigue**	43.61	0.0018	0.17	3.11	2050	431349	<0.0001	4.05	229	3168	Yes	Yes	Yes	6 months	**Moderate comorbid diseases**
**Edema**	24.29	0.1896	-0.07	-1.31	-855	423864	0.2632	-1.19	-62	2972	Yes	Yes	Yes	6 months	
**Apnea**	22.71	0.0036	-0.17	-2.91	-1888	420193	0.0265	-2.22	-123	3024	Yes	Yes	Yes	6 months	
**Acidosis**	7.07	-	-	-	-	-	-	-	-	-	-	-	-	-	**Mild comorbid diseases**
**Hyperglycemia**	3.35	-	-	-	-	-	-	-	-	-	-	-	-	-	
**Esophagitis**	2.56	-	-	-	-	-	-	-	-	-	-	-	-	-	
**Hypercholesterolemia**	1.1	-	-	-	-	-	-	-	-	-	-	-	-	-	
**Hyperhomocysteinemia**	0	-	-	-	-	-	-	-	-	-	-	-	-	-	

**Table 2:** Widespread monthly RSV of GT time series seasonal analysis of the core DSN diseases (Jan 2004 to Dec 2016) without the reference term. (Complete raw data is given in S2 Table).

Seasonal Comorbid Lifestyle diseases (SCLD) of core DSN	Average RSV	Mann-Kendall Trend Test					Seasonal Mann-Kendall Trend Test			
		p-value	tau	z	S	varS	p-value	z	S	varS
**Asthma**	59.09	<0.0001	-0.48	-8.68	-5658	424575	<0.0001	-8.98	-505	3149
**Obesity**	41.71	<0.0001	-0.71	-12.95	-8446	425435	<0.0001	-15.97	-906	3212
**Hypertension**	57.56	<0.0001	-0.64	-11.69	-7620	424882	<0.0001	-13.43	-758	3178
**Fibrosis**	53.76	<0.0001	-0.53	-9.74	-6348	424934	<0.0001	-11.52	-650	3173
**Fatigue**	72.01	0.0010	0.18	3.29	2144	424894	<0.0001	4.13	234	3183
**Edema**	76.26	0.2155	-0.07	-1.24	-808	424478	0.3453	-0.94	-54	3154
**Apnea**	67.82	0.0121	-0.14	-2.51	-1633	423451	0.0881	-1.71	-97	3168
**Acidosis**	75.31	<0.0001	-0.22	-3.96	-2580	424853	<0.0001	-5.6	-315	3154
**Hyperglycemia**	73.01	0.0124	0.14	2.5	1632	425133	<0.0001	3.83	217	3175
**Esophagitis**	62.1	0.0184	-0.13	-2.36	-1537	424163	0.0231	-2.27	-129	3176
**Hypercholesterolemia**	12.42	<0.0001	-0.21	-3.9	-2531	421592	0.0006	-3.41	-192	3137
**Hyperhomocysteinemia**	35.2	<0.0001	-0.46	-8.49	-5535	425072	<0.0001	-8.54	-484	3196

**Fig 12:**
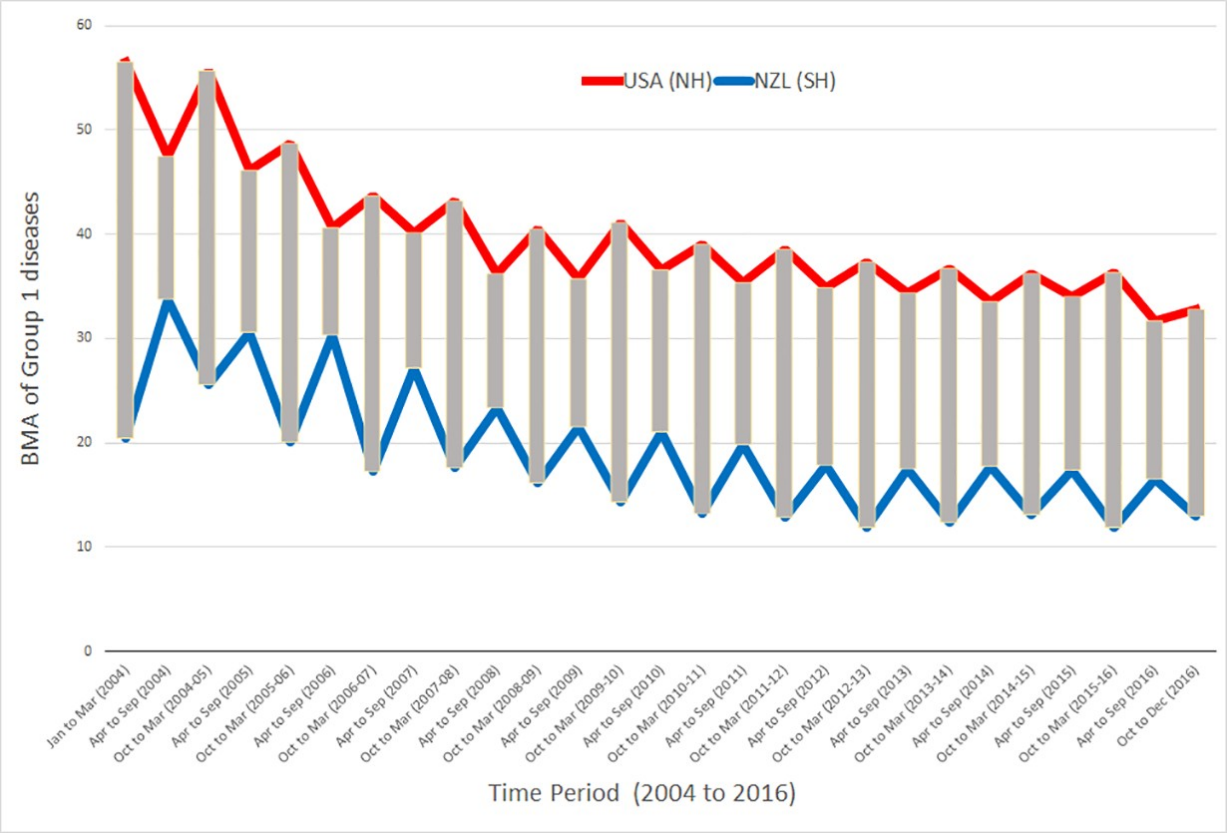
Season wise BMA of group 1 diseases for USA (Red in color) and NZL (Blue in color). The X-axis time period (2004–2016) of each year divided into the window size of 6 months and labelled as April (APR) to September (SEP) and October (OCT) to March (MAR). The Y-axis marked with BMA of Group 1 diseases for the 6 months window size. Please note that USA and NZL seasonal search patterns were juxtaposed (vertical gray bars).

Using obesity as the benchmark keyword, the month wise varying RSV from USA and NZL for group 1 diseases (including obesity) were analyzed for seasonal trends in the 2004–2016 period. The seasonal Mann-Kendall and auto correlation analysis of month wise RSV from both the countries revealed positive seasonal trends and 6 months periodicity respectively in most of the group 1 diseases (Table 3). The BMA of the group 1 diseases from the same periods were combined for USA and NZL separately. The BMA scores revealed that at any point of time USA and NZL have completely opposite search trends for group 1 diseases (Fig 12).

**Table 3:** The monthly RSV of GT time series seasonal analysis of USA (NH) and NZL (SH) for Group 1 diseases (Jan 2004 to Dec 2016) with obesity as the reference term. (Complete raw data is given in S3 Table).

Countries	Group 1 Diseases	Seasonal Mann Kendall test				Auto- correlation	Periodicity (Months)
		z	p-value	S	varS		
**USA (NH)**	**Asthma**	-1.73	0.083	-98	3135.33	No	6,3
	**Obesity**	-14.94	<0.01	-847	3206.33	Yes	6,3
	**Hypertension**	-11.09	<0.01	-621	3127.67	Yes	6,3
	**Fibrosis**	-9.38	<0.01	-522	3106.67	Yes	6,3
**NZL (SH)**	**Asthma**	-4.60	<0.01	-259	3143.67	No	6,3
	**Obesity**	-12.27	<0.01	-690	3170.67	Yes	6,3
	**Hypertension**	-6.68	<0.01	-373	3101.67	No	6,3
	**Fibrosis**	-3.47	<0.01	-192	3044.67	No	3

### Weekly google seasonal trend analysis

The RSV seasonal trends of the query diseases may vary depending upon the selection time interval. To avoid this, weekly RSV of the group 1 diseases were collected for the 2004–2016 period without any benchmark diseases. Furthermore, the group 1 diseases were subjected to Mann-Kendall and seasonal Mann-Kendall analysis. Both the analysis showed strong seasonal trends in the group 1 diseases, also supported by LOESS seasonal decomposition analysis (Table 4). Further, seasonal decomposition analysis by TBATS revealed high seasonal trend in obesity even in the noisy weekly RSV of the four diseases. The optimal shape alignments were also achieved among the group 1 diseases weekly RSV (S4 Table). All these results again strongly suggested that group 1 diseases maintain widespread seasonal comorbid trend.

**Table 4:** Widespread weekly time series seasonal analysis of life style diseases (Jan 2004 to Dec 2016). (Complete raw data is given in S4 Table).

Severe Comorbid Diseases	Average RSV	Mann-Kendall Trend Test					Seasonal Mann-Kendall Trend Test				Seasonal Decomposition	
		p-value	tau	z	S	varS	p-value	z	S	varS	TBATS	LOESS
**Asthma**	84.72	<0.0001	0.13	4.89	28621	34351640	<0.0001	10.79	1262	13665	No	Yes
**Obesity**	74.86	0.0002	0.09	3.61	21225	34382780	<0.0001	12.2	1426	13688	Yes	Yes
**Hypertension**	85.21	<0.0001	0.16	6.15	36025	34355190	<0.0001	14.13	1652	13655	No	Yes
**Fibrosis**	68.48	<0.0001	-0.11	-4.24	-24854	34381480	0.004	-2.88	-339	13808	No	Yes

## Discussion

A fundamental question in biology and medicine is to what degree the seasonality is related to the manifestation of human disorders, a hypothesis that we aimed to test in the present work. We find: (i) HA diseases and disorders with similar signs and symptoms reduced into the core DSN (12 diseases); (ii) RSV time series analysis revealed that most of the core DSN diseases (except edema) have significant seasonal trends; (iii) Among the core DSN diseases, the public interest in asthma, hypertension, obesity, and fibrosis diseases exhibit strong global seasonal comorbid trend in human population. The merits of each findings, limitations, and implications are discussed below.

### Seasonal (sensitive) comorbid lifestyle diseases (SCLD) network

**Fig 13:**
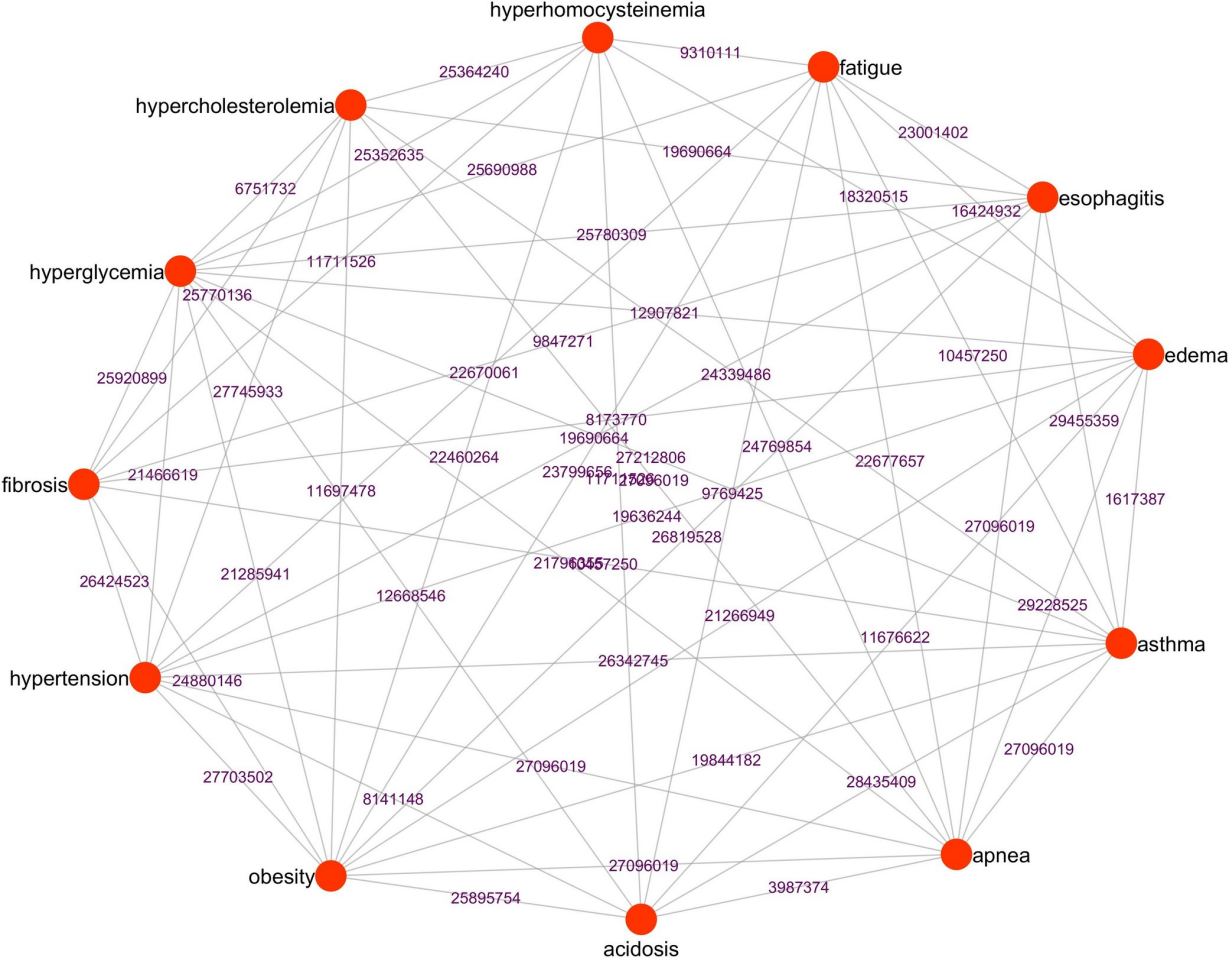
Network view of comorbid association reported in the literature among different diseases of the core DSN. The connection between the different diseases nodes were labelled with their corresponding PubMed Identification number (PMIDs). The complete reference of the labelled PMIDs is given in the **S5 Table**.

The DSN is derived from many clinical and systemic studies of major HA disorders and diseases. The core DSN renders a dense network of highly similar signs and symptoms reveal their strong comorbid association. Several clinical evidences support the strong comorbid association of the core DSN (Fig 13). The core DSN is exploited further to study their shared underlying disease etiologies. There are several clinical evidences that support that the major common etiology of core DSN is seasons along with lifestyle factors such as biological clock, inadequate exercise, and bad diet, which further contribute to their disease progression and activation (Table 5). Based on these findings, we named the “core DSN” as “seasonal (sensitive) comorbid lifestyle diseases (SCLD) network”.

**Table 5:** List of clinical studies (PMIDs) reported the seasonal and life style factors impact on etiology of the core DSN. Please note that this list is not exhaustive and only includes some of the frequently cited reference PMIDs. The citation numbers of each PMIDs as on June 2018 from PubMed is given in the bracket.

Diseases/Symptoms	Seasonal (PMID)	Lifestyle (PMID)
**Edema**	11420189 (44)	5695605 (6)
**Hypertension**	8301109 (50), 22688260 (27), 7074993 (23), 15331861 (8), 20859042 (6)	28775806 (1)
**Asthma**	10899242 (22), 26922932 (4)	22555908 (3), 27238168 (1), 29221919
**Fatigue**	8064638 (1), 26846791	18562170 (15), 11858191 (10), 19812026 (7), 20886027 (3), 17660146 (3)
**Apnea**	22700779 (6)	24082315 (27), 28833858
**Obesity**	16154393 (13)	22591544 (20), 26911589 (5)
**Fibrosis**	18689582 (11)	29045951, 28452727, 29479443
**Acidosis**	26265754	22935845 (12), 22853725 (5)
**Hyperglycemia**	28173630	15589017 (14), 18301331 (11)
**Hypercholesterolemia**	15111372 (44), 3315294 (14), 1482576 (4)	16144549 (11)
**Hyperhomocysteinemia**	9550566 (7)	11447048 (19), 15739593 (2)
**Esophagitis**	26059636 (4), 26235409 (2)	19360912 (23), 22554226 (16)

One of the key objectives in the identification of comorbid diseases is to find a common pathological process of significant clinical importance [57]. Interestingly, the major common pathological process of SCLD flares turned out to be the onset of hypoxic state during their patho physiological process. For example, asthma [58,59], fibrosis [60], apnea [61–63], edema [64–66] and obesity [67,68] create endogenous hypoxic state in lungs, which leads to the disruption of energy supply functions and plastic processes. This lung hypoxic environment increases pulmonary hypertension [69–71] in turn leads to fatigue [72–74], respiratory acidosis [75] and lethal esophagitis [76] in certain maladapted population. Unlikely, to our knowledge, none of the clinical studies addressed the onset of hypoxic state in hypercholesterolemia [77–79], hyperhomocysteinemia [80–83], and hyperglycemia [84–86] patients disease progression. Surprisingly, all the three disorders impair endothelium-dependent vasodilatation by reducing the key hypoxia responsive molecule nitric oxide (NO) production [87]. Overall, the onset of hypoxic state during the disease progression appears to be shared by SCLD. This predicted common pathological process is of clinical importance in the sense that inhaled NO is proposed as a long-term therapy or used as a rescue therapy to some of the SCLD patients [88–91]. Furthermore, individual genetic makeup is one of the major factors that determines an individual’s susceptibility to seasonal changes by invoking complex patho-physiological pathways [92,93].

### Classification of SCLD with benchmark

Several studies have successfully correlated the Google RSV with disease prevalence and their seasonal trend with diseases progression [94,95]. For the first time, a benchmark disease such as obesity based RSV is used to classify the SCLD into severe, moderate, and mild categories. First, based on high RSV, the present study classifies hypertension, obesity, asthma, and fibrosis diseases as the most severe widespread seasonally associated SCLD. In other words, their high RSV indicates that a majority of the human population is more severely subject to seasonal changes in these diseases. Their RSV pattern shape alignments further indicates their strong widespread high bi-seasonal comorbid in the human population. These disease’s high seasonal severity and comorbidity is becoming a major health issue in most of the countries around the world [96]. Second, based on the moderate RSV, the present study classifies edema, apnea, acidosis and fatigue as the moderate SCLD diseases. Several clinical studies indicate the presence of bi and quarterly seasonal variations in them [97–99]. Even though they have the moderate average RSV widespread, in certain countries, the average RSV of the disease terms edema (South America, Italy, Spain, Indonesia, and Lithuania), and apnea (Venezuela, Italy, Spain, and Turkey) is more than the severe SCLD. This may be due to the influence of the language bias in these disease search term RSV. Finally, the present study classifies hypercholesterolemia, hyperglycemia, hyperhomocysteinemia, and esophagitis as mild SCLD diseases/disorders with bi and quarterly seasonal variations. The results, also supported by several clinical studies, demonstrate the seasonal variation of lipid and glycemic levels in blood and serum from the hypercholesterolemia and hyperglycemia patients respectively [100–102]. Although we classify hyperhomocysteinemia as mild SCLD, the evidence of its seasonal variation of blood homocysteine level remains controversial [103]. In tune, the weekly RSV analysis of hyperhomocysteinemia shows no seasonal trend. In USA, a mild but consistent seasonal variation in the diagnosis of esophagitis was observed, which corroborates with the present study [104]. Interestingly, the bi-annual peaks (six months periodicity) in most of the diseases occur in the late winters (October through December) and falls (March through April) (Fig 7). This observation is also of significant clinical importance, especially for the chronic disease asthma. The annual exacerbations rate of asthma follows these two peaks, one in October through December, and the other from March through April and is a well reported global phenomenon [105–108]. Several potentially important SCLD disease risk factors such as seasonal variations in the serum level of insulin, cholesterol, and glucose which also tend to follow the same trend [109].

### Widespread seasonal comorbid rhythm in the severe SCLD

Apart from RSV and high significant seasonal trend, even from the noisy weekly data, we consider obesity as our benchmark disease to predict the comorbidity based on multiple factors. Worldwide obesity (higher BMI values) has nearly doubled since 1980, and current estimates indicate that >1.4 billion adults are overweight or obese [110]. Whilst, there is considerable evidence that obesity is strongly associated with seasonality [111]. In correlation, our study finds the highly significant widespread seasonal trend in the public interest on obesity for the period 2004–2016. However, in modern society, especially those who access internet, are mostly used to living in artificial lighting, heating and air-conditioning systems that considerably reduce the exposure of individuals to seasonal (day and light exposure) and environmental changes [112]. Probably, studies also claimed that the extensive use of these artificial aids may develop mismatch between the season and the body clock that may promote obesity [113,114]. Importantly, being overweight or obese leads to higher prevalence of risk association with chronic diseases such as systemic and pulmonary hypertension, chronic kidney diseases, stroke, obstructive sleep apnea, gastroesophageal reflux disease, type 2 diabetes, osteoarthritis etc. [115]. The risk association of these diseases at any given level of obesity varies with ethnicity. For example, Asians have been shown to have a higher absolute risk of diabetes and hypertension and African Americans to have a lower risk of cardiovascular disease than other groups [116]. Similarly, the prevalence of obesity risk associated diseases varies according to geographical location of the country. For example, the relative risk of death associated with diabetes in Mexico far exceeds that in the United States and Europe [117]. Significantly, first time, our study has shown that public interest in major chronic lifestyle diseases such as obesity, asthma, hypertension, and fibrosis follow a similar strong seasonal pattern or seasonal rhythm which is independent of ethnicity and most likely dependent on seasons. For example, October is commonly associated with the season of autumn in USA and with spring in NZL. In October, our study showed the SCLD search patterns of USA and NZL follow totally opposite seasonal trends and supported by clinical studies [118–122]. In this electronic search study, trends of internet user interest on these SCLD determined for 250 regions from seven continents, suggesting that this is a global phenomenon. This predicted pattern of increase in the prevalence of seasonal comorbid association among asthma, hypertension and obesity is highly supported by clinical evidences [123–125]. Even though fibrotic diseases strongly associate with season, their seasonal comorbid association with obesity, hypertension, and asthma is poorly evaluated [10,126,127]. Further studies in this direction could help healthcare providers to design season based strategies for the better management and prevention or efficacy of treatment start at different months of the year to control the seasonal flare.

## Limitations

This study has several limitations in the text mining as well as electronic search (GT) that need to be considered while interpreting the results.

In text mining, the coverage of diseases and symptoms are limited to the abstracts of “PUBMED”, the search terms “high altitude disease”, “high altitude disorder”, “high altitude medicine”, and “High altitude drug” and the search period (Jan 2004-Jan 2017) [128]. In addition, the DO semantic similarity between DSN diseases should be available in the DOSE package [44]. These limits variety of other diseases, and their interactions could be left out in the DSN probably of decisive importance for the generality of results. In the electronic search, our search study revealed the seasonal trends of public interest in the 12 DSN diseases, but not the seasonal trend of the 12 diseases itself. Furthermore, the individual performing the search is not necessarily suffering from the diseases. To validate our predictions, they should be correlated with clinical data. Meanwhile, the demographic characteristics were not available for the users who were performing the search. Besides the GT ability to cover a large geographical area (250 regions from seven continents), 90% of human population living in the northern hemisphere dominate the worldwide RSV seasonal patterns. Moreover, efforts were not made in our study to give special attention to the remaining 10% of human population living in southern hemisphere seasonal changes. In addition, the seasonal patterns were not studied using any language other than English and with a search engine other than Google. Finally, important covariates other than 12 diseases terms affect the development of these 12 diseases or search behaviors could not be assessed.

## Conclusions

Despite several limitations, there are several strengths in this study. Majority of the human population adapts well to these seasonal changes. But significant world human population maladaptive to seasonal changes, and render their body highly susceptible to one or other kind of disorders [129,130]. Less progress is made to classify this highly seasonal sensitive population from the normal population. This is mainly due to the challenge in prioritizing seasonal sensitive diseases from the environmental sensitive diseases and lifestyle diseases. In this direction, the present study has successfully addressed this issue by predicting the SCLD, and indirectly verified them in the world population using Google Search Trends. Furthermore, based on the SCLD seasonal public interest trend, the study also classified them as severe, moderate and mild. To our knowledge, for first time, these results provide a basis to predict and classify seasonal sensitive population. The study also necessitates the need to study these categories of seasonal sensitive population separately, because they are genetically susceptible host for the SCLD flares. The dense semantic similar diseases network of SCLD further reflects the most possible comorbid seasonal sensitive diseases. Further, knowledge in the so called “seasonal sensitive populations” physiological and molecular response to seasonal triggers such as winter, summer, spring, and autumn become crucial to modulate disease incidence, disease course, or clinical prevention.

## Supporting information

S1 Table Worldwide monthly RSV raw data for the period Jan 2004 to Dec 2016 with obesity as the reference term and shape distance matrix for this period. (XLSX)

S2 Table Worldwide monthly RSV raw data for the period Jan 2004 to Dec 2016 without reference term. (XLSX)

S3 Table Monthly RSV of USA (NH) and NZL (SH) for obesity, hypertension, obesity and fibrosis diseases (Jan 2004 to Dec 2016) with obesity as the reference term. The combined RSV for the Group 1 diseases, the season wise window size of six months period and BMA for Group 1 diseases were displayed for both USA (Red) and NZL (Blue).
(XLSX)

S4 Table Worldwide weekly RSV raw data for the period Jan 2004 to Dec 2016 without reference term and shape distance matrix for this period. (XLSX)

S5 Table The complete reference of the labelled PMIDs of the Fig 13. (XLSX)

## Abbreviations

HA: High altitude
GT: Google Trends
AMS: Acute Mountain Sickness
HAPE: High Altitude Pulmonary Edema
DO: Disease Ontology
GT: Google Trends
RSV: Relative Search Volume
MeSH: Medical Subject Headings
PMID: PubMed Identification Number
CTD: Comparative Toxicogenomics Database
IP: Internet Protocol
API: Application Programming Interface
BeCAS: Biomedical Concept Annotation System
TSA: Time Series Analysis
FFT: Fast Fourier Transform
DSN: Disease Similarity Network
FG: Fast Greedy
EB: Edge Betweenness
SG: Spin Glass
WT: Walk Trap
SCLD: Seasonal (sensitive) Comorbid lifestyle Diseases
NO: Nitric Oxide
BMA: Breaking down Moving Average
